# Cotton fiber tips have diverse morphologies and show evidence of apical cell wall synthesis

**DOI:** 10.1038/srep27883

**Published:** 2016-06-15

**Authors:** Michael R. Stiff, Candace H. Haigler

**Affiliations:** 1 Department of Crop Science, North Carolina State University, Raleigh, North Carolina 27695 USA; 2 Department of Plant and Microbial Biology, North Carolina State University, Raleigh, North Carolina 27695 USA

## Abstract

Cotton fibers arise through highly anisotropic expansion of a single seed epidermal cell. We obtained evidence that apical cell wall synthesis occurs through examining the tips of young elongating *Gossypium hirsutum* (*Gh*) and *G. barbadense* (*Gb*) fibers. We characterized two tip types in *Gh* fiber (*hemisphere* and *tapered*), each with distinct apical diameter, central vacuole location, and distribution of cell wall components. The apex of *Gh hemisphere* tips was enriched in homogalacturonan epitopes, including a relatively high methyl-esterified form associated with cell wall pliability. Other wall components increased behind the apex including cellulose and the α-Fuc-(1,2)-β-Gal epitope predominantly found in xyloglucan. *Gb* fibers had only one narrow tip type featuring characters found in each *Gh* tip type. Pulse-labeling of cell wall glucans indicated wall synthesis at the apex of both *Gh* tip types and in distal zones. Living *Gh hemisphere* and *Gb* tips ruptured preferentially at the apex upon treatment with wall degrading enzymes, consistent with newly synthesized wall at the apex. *Gh tapered* tips ruptured either at the apex or distantly. Overall, the results reveal diverse cotton fiber tip morphologies and support primary wall synthesis occurring at the apex and discrete distal regions of the tip.

Cotton fibers are highly elongated and thickened single seed epidermal cells that provide a valuable tool for probing plant cell growth processes. Fiber morphogenesis begins on the day of anthesis and is completed at about 45 days post-anthesis (DPA) as a result of several dynamic stages of cell wall synthesis, beginning with primary wall synthesis as studied here^1^. Cotton fiber from cultivated *Gossypium* species is also the world’s most important textile fiber due to its exceptional length, strength, and secondary wall thickness. Most of the cotton grown worldwide is high-yielding *G. hirsutum* (abbreviated here as *Gh*), but *G. barbadense* (*Gb*) is grown in compatible environments due to its higher quality fiber^2^. Designing strategies for cotton improvement, for example producing higher quality cotton fiber in more widely grown *G. hirsutum*, will be aided by deeper understanding of the cellular controls of fiber morphogenesis including similarities and differences between genotypes.

Plant morphogenesis is strongly regulated by the interplay between isodiametric turgor pressure, cell wall synthesis, and wall extensibility^3^,^4^. The commonly recognized modes of plant cell growth include: (a) isodiametric diffuse growth, or relatively spherical expansion when cell wall synthesis is distributed around the cell and cellulose microfibrils are randomly arranged; (b) anisotropic diffuse growth when cell wall synthesis is distributed across the surface while oriented cellulose microfibrils constrain cell expansion in one or more preferential directions; and (c) tip growth that depends exclusively on apical cell wall synthesis and results in tubular cells with no diametric expansion behind the tip^5^,^6^. The growth mode(s) for cotton fiber were previously poorly characterized.

Domesticated cotton fibers such as those studied here may ultimately become 4 cm long with about 15 to 17 μm average diameter^7^, implying a length to width ratio of greater than 2300 similar to tip-growing pollen tubes^8^. Whether apical cell wall synthesis occurs in cotton fiber has been a persistent question^9^,^10^,^11^. Some features of cotton fiber tips resemble classical tip-growing cells (pollen tubes, root hairs, and/or moss protonema), although even these cells control anisotropic expansion differently and in ways that we only partly understand^12^,^13^. Like some tip-growing cells, *Gh* fiber tips less than 10 μm in diameter had dense cytoplasm and a remote central vacuole^14^,^15^. *Gh* cotton fiber tips also contain annexin and showed evidence of developmentally regulated calcium influx^16^,^17^.

Diverse cell growth modes occur during the prolonged morphogenetic program of cotton fiber. For example, the fiber initials balloon above the epidermal surface via isodiametric diffuse growth and immediately become wider than the base of the cell in the outer epidermal layer^18^. Until the fibers reach about 80 μm long at 2 DPA, fiber elongation occurs through anisotropic diffuse growth while their tips are blunt^19^. Afterwards, tip tapering occurs in *Gh* fibers^15^,^20^,^21^ and *Gb* fibers^20^,^22^. This tapering implies that, unlike tip-growing plant cells, cotton fibers increase in diameter behind the apex. The extent of diametric expansion is regulated in part by transverse microtubules and cellulose microfibrils, a typical feature of anisotropic diffuse growth^23^. Experiments have supported anisotropic diffuse growth occurring in elongating cotton fiber: (a) radiolabeled sugar as detected by autoradiography was incorporated into cell walls at the tip and along the fiber flanks^9^; and (b) application of microtubule or actin antagonists disturbed the normal orientation of newly synthesized cellulose microfibrils along the flanks^24^.

The occurrence of diffuse growth cannot rule out apical cell wall synthesis because more than one zone of new wall synthesis may exist in an elongating cell. For example, the vegetative hyphae of some species of filamentous fungi exhibit both apical and diffuse growth^25^,^26^. Arabidopsis trichome branches have zones of fast, tip-localized elongation and comparatively slower elongation at the branch base, described as “tip-biased diffuse growth” under the control of a specific zone of tip refinement^27^. Simultaneous apical elongation and anisotropic diffuse growth has been proposed to occur in a single cotton fiber^6^,^10^,^11^, although direct evidence for apical cell wall synthesis was lacking. In addition, evidence for diffuse growth in 2 DPA cotton fibers^19^ is often accepted as if it fully defines the cell (*e.g.*,^5^,^28^). To the contrary, the orientation of microtubules and cellulose microfibrils and tip morphology change immediately after 2 DPA^15^,^29^, supporting the possibility of other growth modes occurring in later stages of development.

Here we show variable morphologies and cell wall characteristics in the fiber tips of two cotton genotypes at 4 to 5 DPA. We characterized broad (*hemisphere*) and narrow (*tapered*) tips in widely grown *Gh* fiber, but found only one narrow tip type in *Gb* fiber. Microscopic analysis of the distribution of cell wall components as detected in fluorescent assays showed that the *Gb* tips possessed characteristics found in both *Gh hemisphere* and *tapered* tips, although their cell walls were most similar to the *hemisphere* tips. Furthermore, the cotton fiber apex was enriched in homogalacturonan epitopes associated with cell wall pliability in other cell types. The exposure of living fibers to cell wall degrading enzymes caused *Gh hemisphere* and *Gb* tips to rupture preferentially at the apex. Pulse-chase labeling of cell wall glucans in living cells provided evidence that apical cell wall synthesis occurs in *Gh hemisphere* and *tapered* tips. Cell wall synthesis also occurred in distal zones of the tip, as shown most clearly in *Gh hemisphere* tips. Overall the results support the ability of the cotton fiber cell to control primary cell wall synthesis in discrete zones of the tip, inclusive of the apex and selected distal regions within 85 μm of the apex.

## Results

Here we use terms for cotton fiber similar to those applied to other elongated cells^12^,^30^,^31^. The ‘apex’ refers to the extreme tip, 0–5 μm from the end. The ‘tip’ that we analyzed extends to 85 μm behind the apex, inclusive of ‘distal’ regions behind the apex. The ‘flanks’ refers to areas more remote from the apex that were not analyzed in this study. ‘Apical cell wall synthesis’ is a description of a biological phenomenon and not a synonym for ‘tip growth’, which exclusively describes cells that exhibit only apical expansion.

**Figure 1:**
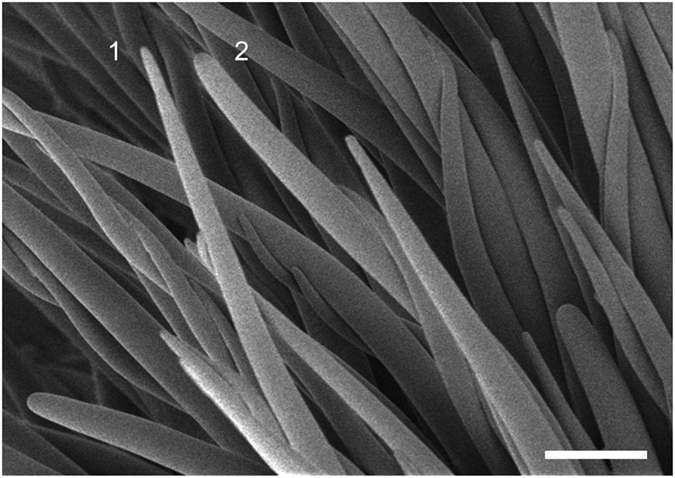
Early elongating fibers of *G. hirsutum* have different tip morphologies as observed by cryo-field emission scanning electron microscopy (cryo-FE-SEM). At 2 DPA, a mixed population of fibers with narrow, pointed tips (fiber 1) or wide, rounded tips (fiber 2) was observed. Different tip morphologies occurred on fibers of similar length. Scale bar = 50 μm.

Viewing young *Gh* fiber *in situ* within the boll by cryo-field emission scanning electron microscopy (cryo-FE-SEM) showed tip variability in the ‘near native’ state (Fig. 1). In this technique a window in the boll wall was cut out immediately before snap-freezing and SEM imaging of the interior fibers^32^. The broad and narrow fiber tips are apparently equivalent to the *hemisphere* and *tapered Gh* cotton fiber tips described earlier, but not characterized further at that time^10^. We characterized similarities and differences of these two *Gh* tip types, along with parallel analyses of *Gb* fiber tips when possible. Our experiments included fibers at 2 to 10 DPA, when both *Gh* and *Gb* fibers grown in parallel were about 6.5 mm long^7^. This allowed comparisons to be made in the context of generally similar outcomes of early fiber elongation in the two species.

### Characterization of tips in *G. hirsutum* and *G. barbadense*

**Figure 2:**
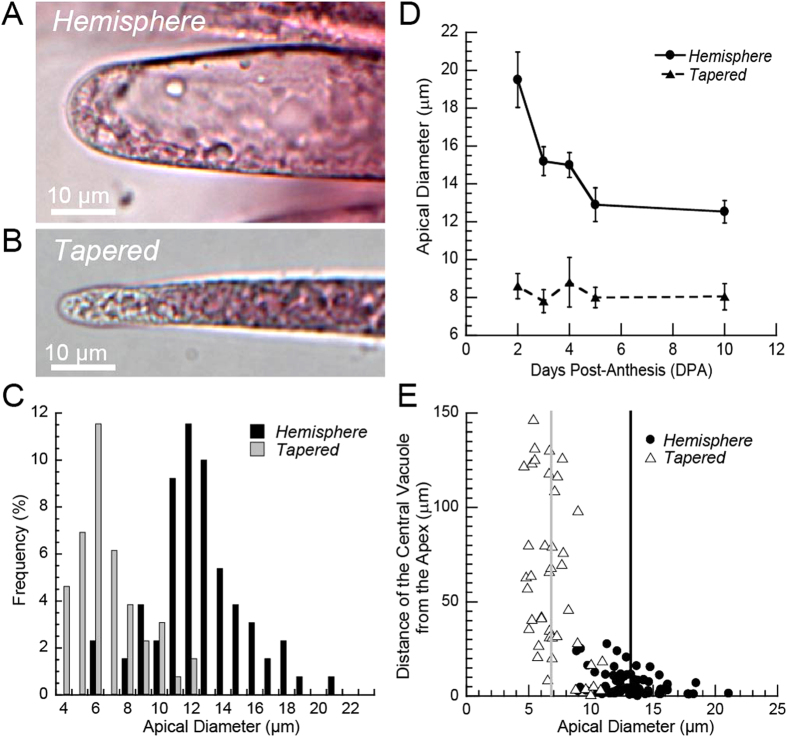
*G. hirsutum* fibers exhibit two tip types during early elongation based on morphology, apical diameter, and central vacuole position. (**A,B**) *Hemisphere* and *tapered* tip morphologies visualized in brightfield microscopy after staining with Ruthenium Red. (**C**) Measurement of apical diameter (at 10 μm from the apex), along with classification of tip shape as *hemisphere* or *tapered*, revealed two partially overlapping, normally distributed, subpopulations of fiber tips (n = 130). (**D**) Fiber tips in the *hemisphere* and *tapered* classes had different average apical diameters at 2–10 DPA (n = 77, 81, 73, 88, and 60, respectively; *P* < 0.0001). The apical diameter of *hemisphere* tips decreased after 2 DPA (*P* < 0.016), whereas no change occurred in *tapered* tips. Error bars represent 95% confidence intervals and statistical analysis was performed using ANOVA and Tukey-Kramer post-hoc test. (**E**) The vacuole was closer to the apex in *hemisphere* tips (6.86 ± 6.54 μm (s.d.); n = 69) as compared to *tapered* tips (60.39 ± 44.19 μm (s.d.); n = 42). The average apical diameter is shown by vertical bars for *hemisphere* (black) and *tapered* tips (grey). The difference in vacuole location between the two tip types was statistically significant (*P* < 0.0001, T-test). Data for (**A–C,E**) were collected at 5 DPA.

The apex of *Gh hemisphere* tips at 5 DPA was rounded with 12.94 μm ± 3.03 (s.d.) average diameter (measured 10 μm back; n = 55 from 3 ovules), whereas the apex of *Gh tapered* tips was more pointed with 7.96 μm ± 2.63 (s.d.) average diameter (n = 33 from 3 ovules) (Fig. 2A,B). The mean apical diameters in *Gh hemisphere* and *tapered* tips were statistically different (*P* < 0.0001, T-test). Within the entire population, there was a bimodal distribution of apical tip diameters (Fig. 2C). The average *hemisphere* apical diameter decreased from 2 to 5 DPA (*P* < 0.016), but remained larger than in *tapered* tips (Fig. 2D). The substantially wider apices of *hemisphere* tips at 2 DPA probably results from measuring many cells that were still in the first phase of anisotropic diffuse growth just after fiber initiation^19^. In contrast, there was no change in the mean apical diameter of *tapered* tips between 2 to 10 DPA. The average distance of the central vacuole from the apex varied with the tip type, with mean values of 6.86 μm or 60.39 μm for *hemisphere* or *tapered* tips, respectively. More variability in vacuolar location occurred in *tapered* tips (Fig. 2E; Supplementary Fig. S1). The percentage of *tapered* tips decreased during early elongation, from a maximum of 71.4% at 2 DPA to 45.8% at 3DPA. From 3 to 10 DPA, the percentage of *Gh hemisphere* or *tapered* tips remained statistically the same, as characterized in fibers of approximately the same length on three ovules at each DPA (Table 1).

**Figure 3:**
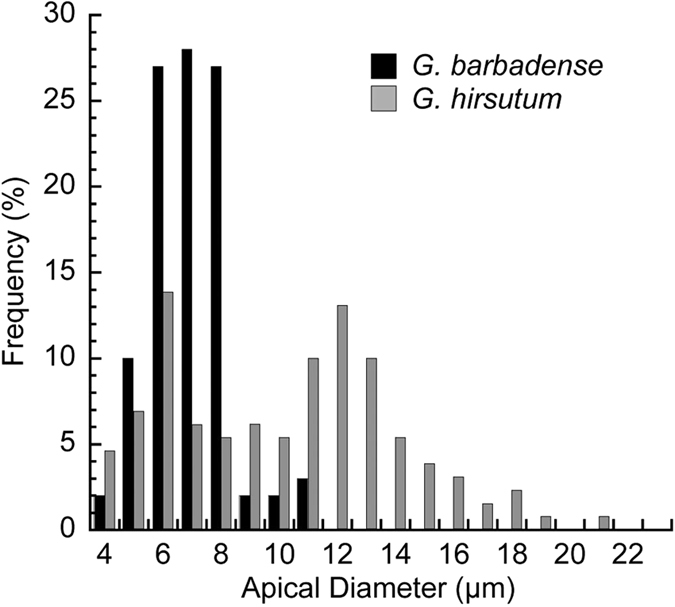
*G. barbadense* (*Gb*) fibers had only one narrow tip morphology at 5 DPA. The distribution of apical diameters (10 μm from the apex) is shown in 1 μm increments (n = 60). Data for *Gh hemisphere* and *tapered* tips from Fig. 2C were combined and overlaid for comparison.

To expand our understanding of cotton fiber tip diversity, we analyzed the tips of *Gb* cv PhytoGen 800, which we have previously compared to *Gh* cv Deltapine 90 in other studies aimed at uncovering potential controls of the higher quality of *Gb* fiber^7^,^33^. Only one narrow tip type was observed in the *Gb* fiber (Fig. 3) with 7.45 μm ± 1.31 (s.d.) average apical diameter (n = 60 from 3 ovules), similar to the 7.96 mean apical diameter of *Gh tapered* tips. The average distance of the central vacuole from the *Gb* fiber apex (34.71 ± 22.29 μm; mean ± s.d., n = 21) was intermediate between and significantly different (*P* < 0.01) from the values for *Gh hemisphere* and *tapered* tips.

### Polysaccharide distribution is consistent with pliable cell walls at the apex of cotton fibers

**Figure 4:**
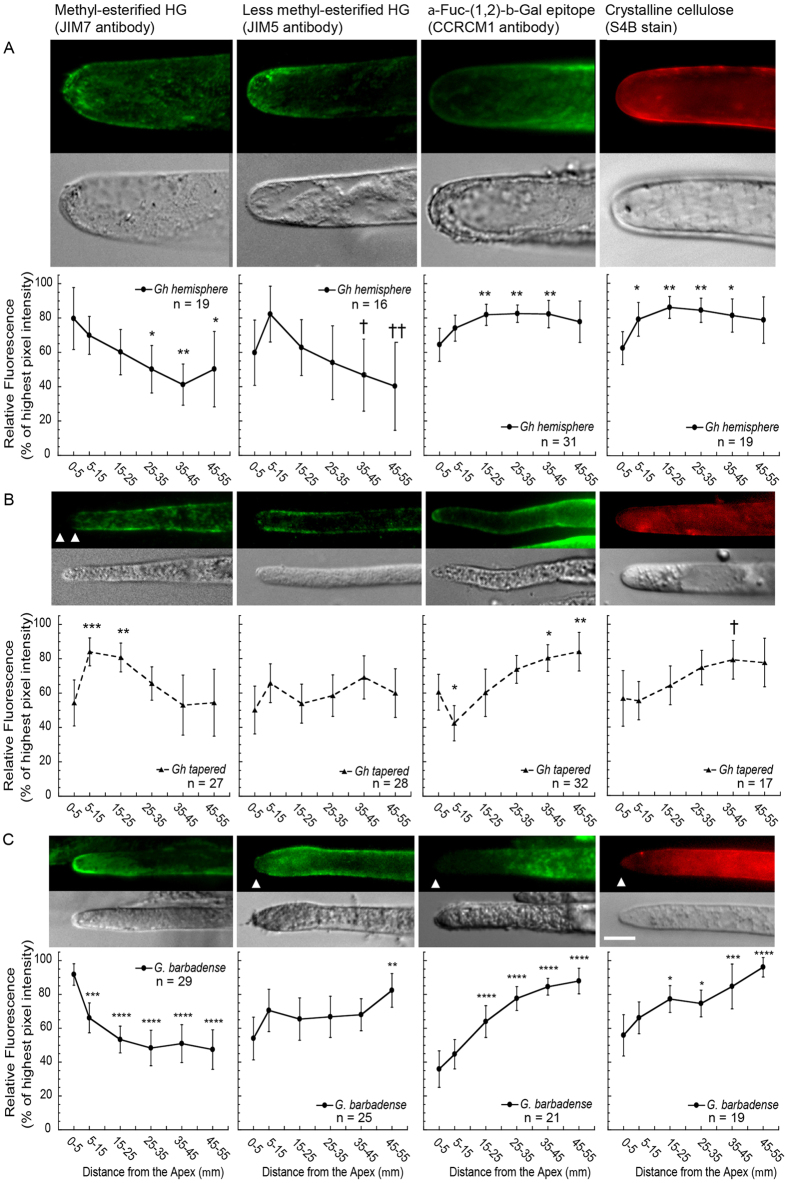
Distribution of characteristic epitopes of homogalacturonan (HG) and xyloglucan (XG) and of cellulose in diverse 4 DPA tips. Cell wall probe names are indicated at the top and the X-axis label is indicated at the bottom of each column. Fluorescent (maximum Z-projections) and brightfield images are shown for representative tips, whereas average values for the tip population are graphed. Significant differences are typically comparisons of each region with the apex (0–5 μm) as indicated by one to four asterisks (*P* ≤ 0.05, 0.01, 0.001, or 0.0001, respectively). Alternatively, one or two daggers indicate significance compared to the 5–15 μm region (*P* ≤ 0.05 or 0.01). (**A**) *Gh hemisphere* tips: HG with relatively high methyl-esterification was enriched near the apex, with lower levels at 25–55 μm in the distal region. HG with relatively low methyl-esterification was also enriched near the apex, and then decreased in the 35–55 μm regions. The α-Fuc-(1,2)-β-Gal epitope was depleted at the apex compared to the 15–45 μm regions. Cellulose was lowest at the apex and higher in the 5–45 μm regions. (**B**) *Gh tapered* tips: HG with relatively high methyl-esterification was depleted at the apex as compared to the 5–25 μm regions. The average amount of HG with relatively low methyl-esterification did not change with distance from the apex. The α-Fuc-(1,2)-β-Gal epitope was lower at 5–15 μm but significantly higher in the 35–55 μm regions as compared to the apex. Cellulose was lowest near the apex and significantly higher in the 35–45 μm region. (**C**) *Gb* tips: HG with relatively high methyl-esterification was most abundant at the fiber apex. The average content of HG with relatively low methyl-esterification was significantly different from the apex only at 45–55 μm. The α-Fuc-(1,2)-β-Gal epitope and the cellulose content were both significantly higher at 15–55 μm compared to the apex. Scale bar = 10 μm applies to all images. Values were graphed at the midpoint of the region measured. Error bars: 95% confidence intervals for (n) fibers analyzed.

We analyzed the average relative abundance of three cell wall epitopes and cellulose in different zones of three types of fiber tips. Figure 4A–C show microscopic and quantitative results for *Gh hemisphere* tips, *Gh tapered* tips, and *Gb* tips, respectively. We hypothesized that cell wall composition would change within the cotton fiber tip region, as occurs in pollen tubes and root hairs. To test this hypothesis explicitly, the graphs display the values in the distal regions of the cotton fiber that are significantly different (according to *p*-values in the figure legends) from the 0 to 5 μm apical value, with two exceptions as described in the Fig. 4 legend. The graphs display the average relative fluorescence in each region as normalized to the highest value in each cell, thereby demonstrating the change in distribution of each probe target along the fiber tip. They cannot be used to compare the absolute amounts of different cell wall components in the fiber tip.

The monoclonal antibodies JIM7 and JIM5 recognize epitopes within HG with higher and lesser degree of methyl-esterification, respectively^34^, and they have been frequently used in analyses of pollen tubes and root hairs (*e.g.*,^31^,^35^,^36^,^37^). More highly esterified HG is less involved in calcium-mediated cross-linking^38^ and remains more pliable to support apical expansion in these classical tip-growing cells. The apex of *Gh hemisphere* tips was enriched in HG with relatively high methyl-esterification, with lower amounts in the 25 to 55 μm distal regions (Fig. 4A). HG with lower methyl esterification was also more abundant near the apex (5–15 μm) than in distal regions. For *tapered* tips, the apex had less HG with high methyl-esterification compared to the 5 to 25 μm region (Fig. 4B). In contrast, the average amount of HG with lower methyl-esterification did not change throughout the *tapered* tips, although 68% of them showed very low apical fluorescence (Supplementary Fig. S2).

Both the *Gh hemisphere* and *tapered* tips had higher amounts of the α-Fuc-(1,2)-β-Gal epitope remote from their apices (Fig. 4A,B) as detected by the CCRC-M1 antibody. This epitope is frequently found within a side group of xyloglucan (XG)^39^. For *hemisphere* tips, the α-Fuc-(1,2)-β-Gal epitope was enriched in the 15 to 45 μm region as compared to the apex. In *tapered* tips, the 35 to 55 μm distal region had the most α-Fuc-(1,2)-β-Gal epitope. The α-Fuc-(1,2)-β-Gal epitope was detected only after enzymatic digestion of pectin (Supplementary Fig. S3), whereas detection of HG epitopes did not require any other cell wall component to be removed first. These results are consistent with electron microscopic immunocytochemistry results, which showed that young *Gh* fibers have an approximately 200 nm thick HG-rich ‘outer pectin sheath’ whereas the cell wall closer to the plasma membrane is enriched in the α-Fuc-(1,2)-β-Gal epitope typical of fucosylated XG and cellulose^40^. The darker staining of the outer layer of the primary cell wall with the uranyl acetate and lead citrate stains commonly used for electron microscopy was consistent with layers with distinct chemical composition. The authors mentioned bilayered epidermal cell walls with asymmetric pectin distribution in other species, while at the same time showing that the synthesis of the outer pectin sheath correlates with cotton fiber elongation^40^.

The fluorophore Pontamine Fast Scarlet 4B (S4B) revealed the distribution of crystalline cellulose^41^ in living fibers, which were assayed to avoid strong S4B-associated cytoplasmic fluorescence in fixed cells (data not shown). The *hemisphere* tips had less cellulose at the apex as compared to the 5 to 45 μm distal regions (Fig. 4A). In the *tapered* tips, cellulose was more abundant at 35 to 45 μm as compared to the 5 to 15 μm region (Fig. 4B). Greater variability at the apex correlated with 61% of *tapered* tips having very low apical fluorescence after S4B staining (Supplementary Fig. S2).

The cell wall characteristics of young *Gb* tips (Fig. 4C) were analyzed in the same way. The *Gb* apex had more HG with higher methyl-esterification as compared to the 5 to 55 μm regions. In contrast, HG with less methyl-esterification, the α-Fuc-(1,2)-β-Gal epitope, and cellulose were lower at the apex of *Gb* fibers, with increases observed at 45 to 55 μm, 15 to 55 μm, or 15 to 55 μm, respectively. These patterns are most like *Gh hemisphere* tips, but differences were observed for the distribution of HG with less methyl-esterification and cellulose. The *Gb* tips had less apical HG with relatively low methyl-esterification as compared to the 45 to 55 μm region, whereas this epitope decreased in the distal regions of *Gh hemisphere* tips. The amount of cellulose continued to increase behind the apex of *Gb* tips reaching the highest level at 45 to 55 μm, whereas similar amounts occurred between 15 to 55 μm in *Gh hemisphere* tips.

### Pulse labeling supports new wall synthesis at the apex and in distal regions of *G. hirsutum* fiber tips

Tinopal LPW was used to pulse-label living 5 DPA *Gh* fibers in order to identify locations of new wall synthesis. This fluorophore highlights both XG and cellulose under imaging conditions similar to ours, and it may also bind to other similar glucans if they are present^41^,^42^. It was used previously to demonstrate localized cell wall synthesis in expanding cultured plant cells^43^ and diffuse growth of flax phloem fibers^44^. If apical cell wall synthesis occurred in cotton fibers, we predicted decreased fluorescence at the apex during growth for 2 to 5 h without fluorophore, as compared to 0 h immediately after the 5 min exposure to the fluorophore. This drop in fluorescence would occur as newly synthesized unstained polymers were integrated into the wall.

**Figure 5:**
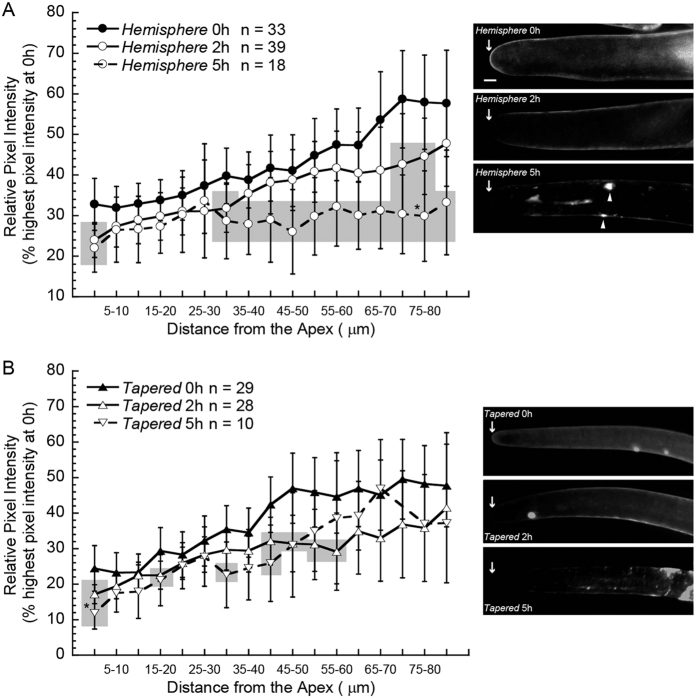
Pulse-labeling with a glucan-binding fluorophore demonstrated apical and distal zones of cell wall synthesis in cotton fiber tips. Living 5 DPA *Gh* fibers attached to ovules were exposed to 0.1% Tinopal LPW and imaged immediately (0 h) or rinsed and grown *in vitro* in the absence of the fluorophore for 2 or 5 h before imaging. Images show maximum Z-projections of representative tips. Grey boxes represent regions at 2 or 5 h that are significantly different from the corresponding region at 0 h (*P* < 0.05, T-test). *Hemisphere* tips (**A**): At 0 h, wall fluorescence was lowest near the apex then increased to reach a plateau 70 μm back. After 2 h, apical fluorescence was 0.7-fold of the 0 h value, and significant reductions also occurred in the 30–35 μm and 70–80 μm distal regions. After 5 h, reduced apical fluorescence as compared to 0 h was again observed, but there was no difference compared to 2 h. Fluorescence was decreased 0.5- to 0.7-fold between 30–85 μm as compared to 0 h. *Tapered* tips (**B**): At 0 h, wall fluorescence was lowest near the apex then increased to reach a plateau 45 μm back. After 2 h, apical fluorescence was 0.7-fold of the 0 h value and significant reductions also occurred in the 15–20 μm and 40–60 μm distal regions. After 5 h, the apical fluorescence was reduced further to 0.5-fold of the 0 h value. Overall, the distal regions of *tapered* tips showed less change than *hemisphere* tips during 2–5 h growth after the Tinopal pulse. Scale bar = 5 μm applies to all images. Arrows show the location of the fiber apex. Measurement regions with bright spots of CFML (arrowheads) were excluded from quantitative analyses. All images were acquired under the same conditions and intensity data were normalized to the highest pixel intensity at 0 h within the population of either *hemisphere* (**A**) or *tapered* tips (**B**). Values were graphed at the midpoint of the region measured. Error bars represent 95% confidence intervals.

We were unable to establish a system for long-term live-cell imaging of cotton fibers because the elongation rate dropped rapidly, making it impossible to monitor processes related to normal elongation. This problem occurred despite testing three solutions to keep fibers hydrated on the microscope stage: Beasley and Ting tissue culture medium (BT^45^), phosphate buffered saline, or water. Therefore, we used the well-established system for growing aerial *Gh* fibers on the surface of ovules floating on BT tissue culture medium^45^. At 5 DPA, ovules with attached fiber were excised from the ovary, briefly immersed in Tinopal LPW to stain the existing cell walls, briefly rinsed to remove unbound fluorophore, and then floated for 2 or 5 h on fluorophore-free BT medium. In the unstained control, the tips of untreated fibers had no visible autofluorescence (Supplementary Fig. S4). Figure 5 highlights the changes that occurred in each region of the tip of aerial fibers at 2 or 5 h as compared to 0 h (grey boxes), as discussed further below. Supplementary Fig. S5 alternatively highlights the differences in distal regions as compared to the apex for each tip type and observation time. At the beginning of the experiment (0 h), both *hemisphere* and *tapered* tips had lowest Tinopal LPW fluorescence at the apex, with increases in the distal regions until a plateau was reached at 70 μm or 45 μm, respectively (Fig. 5; Supplementary Fig. S5). These results were generally consistent with increasing amounts of cellulose and the α-Fuc-(1,2)-β-Gal epitope typically found in XG behind the apex (Fig. 4). A control experiment performed with dead fibers showed that the observed decreases in fluorescence were associated with cell vitality, *i.e.* cell wall growth, and not to spontaneous diffusion of Tinopal LPW out of the cell wall during the chase period without fluorophore (Supplementary Fig. S6).

First we describe changes in fluorescence over time in the 0 to 5 μm apical region for both tip types. After 2 h growth post–fluorophore treatment, both *hemisphere* and *tapered* tips had lower (0.7-fold) apical fluorescence as compared to 0 h (Fig. 5). After 5 h we again observed less apical fluorescence of *hemisphere* tips as compared to 0 h, but there was no difference compared to 2 h (Fig. 5A). In contrast, the apical fluorescence of *tapered* tips at 5 h was lower than at 2 h, having declined to half (0.5-fold) of the 0 h value (Fig. 5B). These results are consistent with apical cell wall synthesis in both tip types, with longer duration under these experimental conditions for *Gh tapered* tips.

Regarding changes over time in the distal regions as compared to 0 h, at 2 h the *Gh hemisphere* tips showed lower fluorescence in the 30 to 35 μm and 70 to 80 μm regions (0.8-fold of 0 h in both regions; Fig. 5A). By 5 h, the fluorescence in the 30 to 85 μm distal region of the *hemisphere* tips was 0.5- to 0.7-fold of the 0 h values. In contrast, no difference was observed in the 5 to 30 μm region of the *hemisphere* tips for either 2 h or 5 h as compared to 0 h, which is consistent with little or no cell wall synthesis in this location during the experiment. By 5 h, the fluorescence was equal between 0 to 85 μm of the *hemisphere* tips (Supplementary Fig. S5). The average cell wall fluorescence in the distal regions of the *tapered* tips showed less change over time, and the 5 h values were greater than the 2 h values in some locations. Considering both the 2 h and 5 h time points together, statistical analysis supported a decline to 0.6- to 0.9-fold of the 0 h value at sporadic regions between 15 to 60 μm in *tapered* tips (Fig. 5B).

Overall the results support the ability of the fiber cell to control primary wall synthesis in discrete zones of the tip, inclusive of the apex and separate distal regions. We observed no diametric expansion at 2 or 5 h compared to 0 h (Supplementary Fig. S7) or obvious changes in wall thickness or uniformity after pulsed exposure to Tinopal LPW (Fig. 5). Presumably, the new cell wall synthesis is supporting fiber elongation, but this remains to be proven when a robust live cell imaging system for cotton fiber is available.

### Cell wall digestion provided evidence for new cell wall synthesis at the apex of *G. hirsutum hemisphere* and *G. barbadense* tips

**Figure 6:**
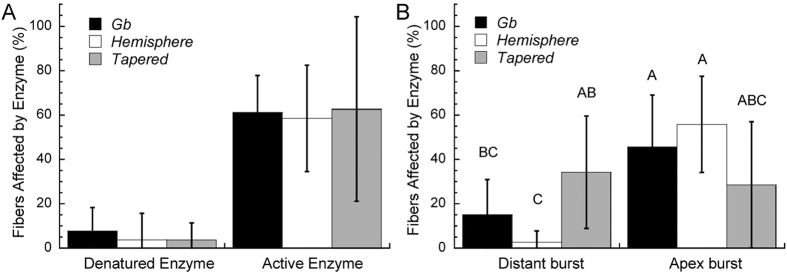
The location of rupture after enzymatic digestion revealed the weakest points in the cell wall of *Gb*, *Gh hemisphere*, and *Gh tapered* tips. After 5 min incubation of living fibers in active cell wall-degrading enzyme (or denatured enzyme as a control), the location of cell rupture was classified as ‘Apex burst’ or ‘Distant burst (>5 μm from the apex)’. (**A**) Active cell wall degrading enzymes caused about 60% of fiber tips to rupture (*P* < 0.001 for all fiber tip types compared to their denatured enzyme control as determined by ANOVA with Tukey’s post-hoc test), with the most variability observed for the fate of *Gh tapered* tips. (**B**) Analysis of the location of fiber rupture showed that bursting occurred most frequently at the apex in *Gb* tips (*P* < 0.05) and *Gh hemisphere* tips (*P* < 0.001) as compared to the frequency of distant bursts. *Gh tapered* tips burst equally at the apex or distantly. Groups of significantly different means, as indicated by different letters, were determined by ANOVA with Tukey’s post-hoc test. In both graphs, error bars represent 95% confidence intervals. N = 4 experiments, inclusive of measurements for 74–80 individual fiber tips.

Apical bursting induced by cell wall degrading enzymes has been attributed to weak new cell wall existing at the apex of tip-growing root hairs and pollen tubes^36^,^46^. We incubated living *Gb* and *Gh* fibers in a cell wall-degrading enzyme mixture containing predominantly pectinase activity, as well as lower cellulase and hemicellulase activity, then categorized the burst locations as apical (less than 5 μm from the apex) or distant (greater than 5 μm from the apex; see images in Supplementary Fig. S8). For all three tip types, about 60% ruptured in the presence of active enzymes, whereas the majority remained intact in denatured enzyme control treatments (Fig. 6A). The majority of *Gh hemisphere* and *Gb* fibers ruptured preferentially at the cell apex (Fig. 6B), whereas *Gh tapered* tips ruptured equally at the apex and distantly.

## Discussion

The results reported here illustrate a previously uncharacterized variation in the tips of young elongating cotton fibers. The *hemisphere* and *tapered* morphologies of *Gh* tips were observed in this study in two cultivars, *Gh* cv Coker 312 (Fig. 1) and *Gh* cv Deltapine 90 (Fig. 2). They can also be recognized in *Gh* cv Maxxa^15^. Therefore, tip diversity is a common feature in modern *Gh* cotton cultivars. The different tip types occurred on fibers of the same length and were not attributable to the co-mingling of tips of a separate population of short *Gh* ‘fuzz’ fibers that initiate at least four days later than the long ‘lint’ fibers^22^,^47^,^48^, forming an undercoat on the seed. In contrast to the two tips types observed in *Gh* fiber, the *Gb* tips were all of one morphological type. Their cell wall polysaccharide distribution was most similar to *Gh hemisphere* tips even though the *Gb* tips had narrow apical diameter like the *Gh tapered* tips.

We cannot currently determine whether *Gh hemisphere* and *tapered* tips indicate two phases of growth of one elongating fiber cell or two distinct ‘lint’ fiber populations on the same seed. However, there are indications that tip morphology can change in one fiber. For example, the tip tapering that occurs at the end of the fiber initiation phase^15^,^21^,^49^ likely explains the occurrence of the highest percentage (71%) of *Gh tapered* tips at 2 DPA (Table 1). The percentage of *tapered* tips declined to about 46% at 3 DPA, suggesting a conversion of some *tapered* tips to *hemisphere* tips that will need to be tested in future live cell imaging experiments once such a system is developed for cotton fiber.

Experiments on living cells provided evidence for apical cell wall synthesis in *Gh* and *Gb* fibers. For the *Gh hemisphere* tips, we observed: (a) reduced Tinopal fluorescence at 0 to 5 μm during growth without the fluorophore (Fig. 5A); and (b) bursting most frequently at the apex in the presence of cell-wall-degrading enzymes (Fig. 6). The reduced fluorescence is consistent with new unstained cell wall polymers added to the apical cell wall after pulse-staining at 0 h. Apical bursting in similar cell wall degradation experiments on root hairs and pollen tubes was attributed to the rapid degradation of newer, more extensible cell wall at the apex^36^,^46^. *Gh tapered* tips also showed evidence of apical wall synthesis that continued for 5 h in culture after Tinopal staining (Fig. 5B), and many of them burst at the apex in the cell wall-degradation assay (Fig. 6). The Tinopal assay could not be performed with *Gb* fibers because they do not elongate well on ovules cultured using the procedures that are routine for *Gh* ovules (data not shown). However, the *Gb* tips also burst preferentially at the apex in the cell wall degradation assay. Without a robust live cell imaging system for elongating cotton fiber, only a speculative estimate of apical growth rate can be made. The Tinopal fluorescence of both *hemisphere* and *tapered* apices (0–5 μm region) decreased 30% during a 2 h pulse. Assuming that this reduction arises due to 30% (1.5 μm) increased length of the 5 μm apical region as new cell wall material is deposited, the calculated apical elongation rate of the aerial fibers during the pulse-chase experiment is 0.75 μm/h.

The apices of both *Gh hemisphere* and *Gb* tips were enriched in HG with relatively high methyl-esterification, which also occurs in tip-growing pollen tubes to promote apical cell wall extensibility^8^,^31^,^36^. *Gh tapered* tips did not show apical enrichment (at 0–5 μm) of HG with relatively high methyl-esterification despite evidence for the occurrence of apical cell wall synthesis (Figs 4 and 6). However, over 60% of *Gh tapered* apices showed very weak fluorescence associated with cellulose and less methyl-esterified HG (Supplementary Fig. S2), which is also lower in pollen tube apices^31^. A proportion of the *Gh tapered* tips could have wall properties that facilitate apical expansion somewhat differently than *Gh hemisphere* tips and other highly elongated plant cells. Further experiments are needed to fully understand the biophysical and morphogenetic implications of cell wall diversity at the cotton fiber tip. The existence of HG in cotton fiber tips undergoing apical cell wall synthesis may help to explain the observations that the addition of pectin precursors (UDP-rhamnose and UDP-galacturonic acid) increased cotton fiber length *in vitro* and cotton homologs of pectin biosynthesis genes restored normal root hair length when over-expressed in mutant Arabidopsis plants^50^.

The Tinopal experiments provided evidence for cell wall synthesis in the 35 to 85 μm distal regions of *Gh hemisphere* tips (Fig. 5A). The data also supported a lesser extent of cell wall synthesis in sporadic distal regions of the *Gh tapered* tips (Fig. 5B). These results are consistent with early autoradiography analysis of the cell walls of 4 and 11 DPA fibers of *G. arboreum*^9^, a diploid species related to one of two ancestors of modern tetraploid *Gh* and *Gb* cottons^51^. The pattern of incorporation of radiolabeled sugar supported preferential cell wall synthesis at the tip, inclusive of the apex and about 100 to 200 μm of the distal region, while a lower level of radiolabel incorporation also occurred all along the fiber flanks^9^. For a speculative estimate of distal elongation rate in the *hemisphere* tips, we used the average fluorescence values between 30–85 μm to determine a 32% average decrease at 5 h as compared to 0 h. Assuming that this decrease reflects 17.6 μm of additional length during 5 h, the calculated elongation rate is 3.52 μm/h for this 55 μm distal region of the tip during the pulse-chase experiment.

In contrast to evidence for new cell wall synthesis in the 35 to 85 μm distal region, the 5 to 30 μm region just behind the apex of *Gh hemisphere* tips showed no evidence of primary cell wall synthesis in the Tinopal pulse-staining experiments (Fig. 5A). These data provide evidence that the cotton fiber cell can regulate different zones of primary wall synthesis in the tip region. Conceptually similar conclusions about the zonal regulation of primary wall synthesis, with different spatial details, were obtained for trichome branches in which analysis of fiducial markers supported selective cell growth in the approximately 8 to 30 μm distal regions behind the apex while little apical or basal expansion occurred^27^.

We previously discussed a ‘cell wall toolbox’ that plant cells have used in different ways to accomplish their individualized morphogenetic programs^52^. Cotton fiber uses this ‘toolbox’ flexibly to control several phases of morphogenesis, including diametric expansion behind the apex (Fig. 1) that does not occur in tip-growing plant cells. Correspondingly, the cell walls of the cotton fiber tip have unique features. In all three cotton fiber tip types examined, both the α-Fuc-(1,2)-β-Gal epitope and cellulose increased in the distal part of the tip as compared to the apex. Given that the α-Fuc-(1,2)-β-Gal epitope is frequently found in XG, this pattern is consistent with a role of XG-cellulose interactions in regulating cell wall biomechanical properties^53^. In contrast, the α-Fuc-(1,2)-β-Gal epitope is distributed uniformly in the tips of both pollen tubes and root hairs^31^,^46^. Cellulose is more concentrated at the root hair apex, whereas pollen tubes can have a strong or weak presence of apical cellulose, possibly related to temporal oscillation in growth phase^31^,^46^. The pattern observed in the cotton fiber tips is consistent with a more pliable wall at the apex and a stronger wall in the distal region to control diametric expansion (Supplementary Fig. S7).

In tip-growing cells, the abundance of HG with lesser or higher methyl-esterification changes inversely in the tip^31^,^36^. These distributions are mediated by localized activity of pectin methyl-esterases (PMEs) that remove methyl groups of the initially-secreted HG to facilitate wall rigidification through calcium cross-linking of HG polymers behind the apex^35^,^54^. *Gb* tips showed an inverse relationship of relative amounts of the two forms of HG, but this relationship was not observed in either *Gh* tip type (Fig. 4). These contrasting observations parallel the different degrees of HG methyl-esterification between older *Gh* and *Gb* fiber (15–22 DPA)^55^. A pectate lyase (GhPEL) enhances *Gh* fiber length^56^, and it could work behind the apex of *Gh hemisphere* tips to degrade the less methyl-esterified HG preferentially so that this epitope declines in the distal region along with the higher-esterified HG that may diminish due to the pectin methylesterase activity.

### Summary and Questions for Future Research

The data presented here provide evidence that cell wall synthesis in cotton fibers occurs at the apex and in distal regions of the tip. Collectively, the data support a previously hypothesized ‘mixed mode’ of growth for a single plant cell in general^6^ and cotton fiber in particular^10^,^11^. It is likely that dispersed zones of new wall synthesis support elongation in the cotton fiber tip, although this remains to be directly established once a robust system for dynamic live cell imaging of cotton fiber growth becomes available. Despite substantial effort, we were not able to develop a system to sustain elongation in submerged cotton fibers, which is why the Tinopal experiments were done on the aerial fibers of pre-stained floating ovules. If a system for continued elongation of fibers in solution on the microscope stage were available, it would potentially be possible to use soluble styryl dyes to demonstrate specific sites of vesicle fusion in young cotton fibers, as has been useful for demonstration of tip growth in root hairs and pollen tubes^57^,^58^. In addition, further work to develop methods for the use of fiducial markers during long term imaging of living elongating cotton fiber, as recently done for Arabidopsis trichome branches^27^, will be worthwhile.

The data also present many other questions to stimulate future experiments. What is the rate of elongation in the different tip types and in different zones of the tips? In *Gh*, after the post-initiation formation of *tapered* tips, does a single fiber oscillate between *hemisphere* and *tapered* tip morphology throughout elongation? What are the detailed cellular mechanisms that regulate apical cell wall synthesis in early elongating cotton fibers, and how is cell wall synthesis controlled in discrete zones of the tip? Where are pectin-modifying and/or degrading enzymes located in the tip? Does apical cell wall synthesis occur in later stages of cotton fiber development than those characterized here? Do the different *Gh* tip types make different contributions to final fiber length and/or average final fiber diameter? In *Gb*, does the existence of only one tip type contribute to greater final fiber length and uniformity as compared to *Gh* fiber? Further mechanistic research will increase our knowledge of how plant cells regulate primary cell wall synthesis in different zones and support the design of strategies to improve the quality of commercial cotton.

## Methods

### Plant growth, ovule harvest, and fixation

*Gossypium hirsutum* cv. Deltapine 90 (DP90) and *G. barbadense* cv. PhytoGen 800 were grown as before^32^ in a greenhouse of the NC State University Phytotron. *G. hirsutum* cv Coker312 (C312) analyzed by cryo-FE-SEM was grown in a greenhouse as described previously^32^. Ovules were dissected at ages indicated for each data set. Fixation, when used, commenced immediately upon dissection of ovules from the bolls in the greenhouse and continued for 1 h (RT; gentle rocking; HistoChoice, Electron Microscopy Sciences, 64115-01).

### Microscopic characterization of tip morphology

For light microscopy, well-developed ovules from the lower two-thirds of the locule of the bolls of three plants per genotype were dissected at 2 to 5 and 10 DPA. After fixation, ovules were rinsed with dH_2_O (3 × 5 min), stained 10 min with 0.02% w/v Ruthenium Red (Sigma-Aldrich, R2751-1G), and washed (10 min, dH_2_O). Digital images of fibers from the ovule chalazal end were captured with brightfield optics. For the long fibers found at the chalazal end of the ovule^59^, apical diameters were measured 10 μm back from apex using ImageJ 1.46a^60^. Methods for cryo-FE-SEM have been previously described^32^.

### Microscopic imaging of cell wall components

Fibers attached to the ovules were analyzed. Fluorescence microscopy and image processing was performed with a system including an IX81 inverted microscope, an IX2-DSU disk scanning unit (Olympus), an ORCA-ER CCD camera (Hamamatsu Photonics), and SlideBook versions 4.2 or 5.0 software (3i-Intelligent Imaging Innovations). Binning (2 × 2) was used to increase the signal to noise ratio. The Alexa Fluor 488 fluorophore was visualized with excitation S492/18 nm and emission S535/40 nm filters in confocal mode. S4B fluorescence was monitored with 572/23 nm excitation and 630/60 nm emission filters in successive Z-planes using widefield optics to reduce image acquisition time from a living cell. Tinopal LPW fluorescence was visualized in living cells as for S4B except the excitation 350/50 nm and emission 465/30 nm filters were used. Paired images were often taken with differential interference contrast (DIC) or brightfield light microscopy.

For probes of cell wall components, exposure times were optimized for signal detection and fluorescent pixel intensities were normalized internally for each fiber as described in detail below. For Tinopal LPW, all fibers were imaged with a 75 ms exposure time under identical optical conditions so that the fluorescence intensities could be compared within the entire populations of either *hemisphere* or *tapered* tips during this time-course experiment.

Single midplane optical sections showed that all of the probes targeted cell walls while the cytoplasm of intact fibers remained unstained (Supplementary Fig. S9). Most of the fluorescence intensity measurements were made on Z-stacks of multiple optical sections due to the difficulty of capturing a substantial length of the fiber in any one optical plane. The pattern of relative fluorescence was the same when measured from maximum Z-projections or when the midplane was followed through multiple optical sections (Supplementary Fig. S9). Additional details of individual experiments are described below.

For immunological detection of cell wall epitopes, fixed ovules were rinsed in Tris Buffered Saline (TBS, 10 mM Tris, 150 mM NaCl, pH 7.4), transferred to CellSafe Biopsy inserts (Electron Microscopy Sciences, 62327-10), and washed in TBS (3 × 10 min). Primary monoclonal antibodies, JIM7, JIM5, and CCRC-M1 (Complex Carbohydrate Research Center, University of Georgia), were applied at 1:100 dilution in 5 mL TBS containing 1% (w/v) bovine serum albumen (BSA) and 0.02% (w/v) sodium azide (1 h, RT, shaking). After washing (TBS, 3 × 15 min, RT, shaking), secondary antibody [goat anti-rat Alexa Fluor 488 (Life Technologies/Thermo Fisher Scientific Corporation, A11006) for JIM5 and JIM7 or goat anti-mouse Alexa Fluor 488 (Life Technologies/Thermo Fisher Scientific Corporation, A11001) for CCRC-M1] was applied at 1:200 in TBS/BSA/azide (1 h, RT, dark, gentle shaking). After washing (TBS, 3 × 15 min, shaking, dark), fragments of the chalazal end of ovules with attached fiber were mounted in ProLong Gold Antifade Mountant (Life Technologies/Thermo Fisher Scientific Corporation, P36930).

For labeling the α-Fuc-(1,2)-β-Gal epitope with CCRC-M1, pectin was enzymatically digested following published methods^61^. Fixed and washed ovules were transferred to 50 mM sodium acetate buffer, pH 5.0 (15 min) then digested (30 min, gentle shaking) in the same buffer containing 0.5 mg/mL saponin (Sigma-Aldrich, S2149), 0.5 mg/mL pectinase (Sigma-Aldrich, P2401), 0.5 mg/mL pectolyase (Sigma-Aldrich, P3026), 2.5 mg/mL gelatin (Sigma-Aldrich, GB150), and protease inhibitor cocktail (1% v/v; Sigma-Aldrich, P9599). The ovules were washed in TBS (3 × 10 min) before continuing with primary antibody incubation.

For fluorescent detection of cellulose by Pontamine Fast Scarlet 4B (S4B; Sigma-Aldrich, S479896), dissected ovules were laid on a 24 × 50 mm coverslip, which allowed greater working distance on the inverted microscope. The living fibers were wetted with Beasley and Ting medium [BT; 0.5 μM GA_3_, 5.0 μM IAA, pH 5.0^45^, except 120 mM glucose was used as the sole carbon source]. The ovule was covered with a 22 × 22 mm coverslip resting gently over the chalazal end, which prevented ovule movement while allowing the micropylar end to remain aerated. BT medium was wicked away and the space between the coverslips was filled with 0.1% S4B in BT medium. After 5 min, the stain was wicked away. Ovules were rinsed and mounted in fresh BT medium. Images were captured from living chalazal fibers (with cytoplasmic streaming) within 30 min from 6 to 8 ovules per genotype.

Pulse labeling of cell walls with Tinopal LPW provides a means of defining regions of new cell wall synthesis through their reduced fluorescence during a period of growth without the fluorophore^43^,^44^. For pulse-labeling cell wall glucans in living fibers, 5 DPA dissected *Gh* ovules were submerged (5 min, gentle shaking) in filter-sterilized 0.1% (w/v) Tinopal LPW (Ciba-Geigy; a formulation of C.I. Fluorescent Brightening Agent 28, CID 6108780) in sterile dH_2_O, rinsed twice, and imaged immediately (0 h) or incubated (dark, 30 °C, 2 h and 5 h) while floating on BT medium that had been pre-warmed to 30 °C. Ovules were mounted as described for S4B staining, and the tips of living fibers (as shown by cytoplasmic streaming) were imaged. All Tinopal LPW solutions and treatments were protected from light until observation.

In these experiments, only aerial fibers on the top surface of floating ovules were imaged. This minimized the possibility of Tinopal spontaneously diffusing out of the cell wall during the chase period, even in the unlikely event that this would occur for this chemical that is added to detergents as a ‘brightener’ for cotton clothing^62^. In prior uses of Tinopal LPW to analyze cell growth, living cells were submerged for at least 48 h and maintained strong cell wall fluorescence in older cell wall regions^43^,^44^. We demonstrated that Tinopal did not passively diffuse away from cell walls in the cotton fiber tip during 5 h after staining in a control experiment in which ovules were handled the same way except for being killed after staining with a third wash containing 0.02% sodium azide. The fluorescence intensity at 0 h and 5 h was analyzed in the same way as for the living fibers, and it did not change in the dead cells (Supplementary Fig. S6).

### Quantification of fluorescence

Similarly to published methods^31^, fluorescence intensity distribution was determined along the periphery of the cotton fiber tips in maximum Z-projections using SlideBook software. The fiber periphery was traced with the small pencil tool with a 630 nm effective width that covered the 150 to 200 nm width of the primary wall^32^ and its slight broadening at the perimeter of the Z-stacks (Supplementary Fig. S10). No substantial autofluorescence was included on the edges of the trace for any filter set used (Supplementary Fig. S4). Incremental regions of the cell wall were measured so that those with adhering polymers of the secreted cotton fiber middle lamella (CFML) could be discarded from quantitative analysis. For JIM7, JIM5, CCRC-M1, and S4B, measurement regions included the perimeter of the cell apex (0–5 μm back) and 10 μm increments covering a 55 μm distal length of the tip cell wall. When possible in the absence of CFML, an average pixel intensity value was obtained for the two edges of the fiber at each distance from the apex. Images were processed to allow comparisons of fluorescence intensity in different measurement regions as previously described^63^, and the average pixel intensity at each distance from the apex was normalized to the highest value found in each fiber. Finally, an average value for that location was determined in *Gh hemisphere*, *Gh tapered*, or *Gb* tips. The same method was used in the Tinopal experiment on *Gh* fibers except 5 μm long measurement regions were used to include 85 μm back from the apex. Each measurement of pixel intensity was normalized to the highest value in the population of *Gh hemisphere* or *tapered* tips at 0 h in order to allow changes over time to be compared. From 10 to 39 individual tips were analyzed for each cell wall component.

### Cell wall digestion in living fibers

To assess regions of greatest cell wall extensibility by fiber rupture location, living 5 DPA *Gh* and *Gb* ovules with attached fiber were incubated in 1 U/mL of a cell wall degrading enzyme mixture (Sigma-Aldrich, P2401) in BT medium for 5 min, RT. Control experiments were performed using heat denatured enzyme (65 °C, 45 min) in the same concentration. Ovules were then fixed, stained (10 min, 0.02% (w/v) Ruthenium Red), and washed (10 min, dH_2_O) prior to light microscopy. Fiber tips were classified as Intact (no ruptures); Distant burst (rupture >5 μm from the apex); and Apex burst (rupture <5 μm from the apex). A few fibers ruptured both at the cell apex and more distantly, and these were classed as “Distant burst” as the most conservative choice given our hypothesis that the enzymes would cause bursting preferentially at the apex. A total n = 74 to 80 individual *Gh hemisphere*, *tapered*, and *Gb* tips were analyzed within four independent experiments.

### Statistical analyses

Student’s T-tests were performed in Microsoft Excel 2007 and ANOVA was performed using the General Linear Model procedure in SAS 9.2 (Cary, NC). The data in Fig. 4 and Supplementary Fig. S5 were analyzed by ANOVA and Dunnett’s post-hoc test in order to test the hypothesis that the fluorescence intensity in distal regions of the tip would differ from the value at the apex^64^. Significant differences within each column in Table 1 and groups of significantly different means in Fig. 6 were determined by ANOVA followed by Tukey-Kramer post-hoc test. In all tests, significance was determined by *P* ≤ 0.05 and represented using recommended best practices^65^,^66^. In reporting and discussing results, we only assert differences when they are supported by statistical analysis. The *p*-values and statistical tests used are available in each figure legend.

## Additional Information

**How to cite this article**: Stiff, M. R. and Haigler, C. H. Cotton fiber tips have diverse morphologies and show evidence of apical cell wall synthesis. *Sci. Rep.* **6**, 27883; doi: 10.1038/srep27883 (2016).

## Supplementary Material

Supplementary Information

## Figures and Tables

**Table 1:** Percentage of Gh tip types observed at 2 through 10 DPA.

DPA	% of total tips at each DPA	
	*hemisphere*	*tapered*
2	28.6 ± 2.3*	71.4 ± 3.2*
3	54.2 ± 1.5	45.8 ± 0.6
4	66.7 ± 2.0	33.3 ± 4.2
5	62.3 ± 4.5	37.7 ± 3.0
10	50.0 ± 1.2	50.0 ± 1.2

Values represent mean ± s.d, with asterisks indicating statistically different means within each column (*P* < 0.01). For each DPA, n = 3 ovules from different plants and 58–88 total fiber tips were analyzed.
