# Supplementary material for: Cotton fiber tips have diverse morphologies and show evidence of apical cell wall synthesis
Source: Sci Rep. 2016 Jun 15;6:27883. doi: 10.1038/srep27883 (PMC4908599; doi:10.1038/srep27883)
Supplement: Supplementary Information [file srep27883-s1.pdf]

Supplementary Information Online for:

Cotton fiber tips have diverse morphologies and show evidence of apical cell wall synthesis

Michael R. Stiff[1] and Candace H. Haigler[1, 2]
[1]Department of Crop Science and [2]Department of Plant and Microbial Biology, North Carolina State University, Raleigh, North Carolina 27695 U.S.A.

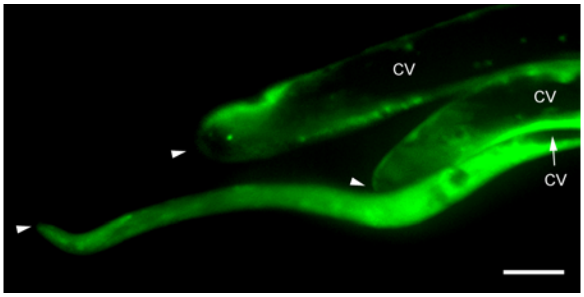

Supplementary Figure S1. Central vacuoles were closer to the apex in living *Gh hemisphere* compared to *Gh tapered* tips. Green fluorescence indicates cytoplasm while the black area within the cell perimeter is the unstained large central vacuole (CV), which is closer to the apex in *Gh hemisphere* tips (top and middle fibers) than in this *Gh tapered* tip (bottom fiber). The vacuolar location in this *tapered* tip is in the middle of the highly variable range observed for the population of *tapered* tips (**Figure 2** in the main text). Living 5 DPA *Gh* fibers attached to ovules were incubated with 25 µM fluorescein diacetate (FDA; a vital stain) in 0.1% DMSO for 15 min in the dark at 30°C, rinsed three times with dH_2O, and then incubated for 10 min in dH_2O. The apex of each cell is noted by an arrowhead. Fibers were visualized with (FITC) excitation (S492/18 nm) and emission (S535/40 nm) filters. Scale bar = 10 µm.

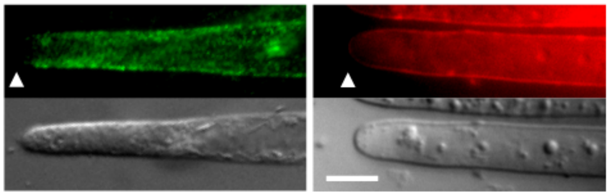

Supplementary Figure S2. Homogalacturonan (HG) with lower methyl-esterification and crystalline cellulose were often undetected in the apex of many *Gh tapered* tips. HG was localized in 4 DPA fibers by immunofluorescence (JIM5 antibody; left panel) and crystalline cellulose was localized by S4B fluorescence (right panel). Many *Gh tapered* tips showed lower JIM5- and S4B-related fluorescence at the apex (68% and 61% of fibers sampled, respectively; n = 28 and 17). Arrowheads indicate the location of the cell apex. Scale bar = 10 μm.

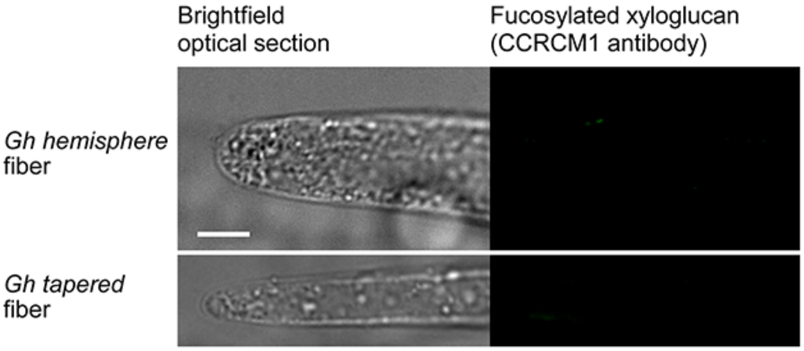

Supplementary Figure S3. Distribution of the CCRC-M1 epitope in 4 DPA *Gh* fiber tips was not observed without pectinase pre-treatment. Fibers fixed in HistoChoice® and without any pectinase pre-treatment (left panel) were probed with the CCRC-M1 antibody, which recognizes α-Fuc-(1,2)-β-Gal commonly found in xyloglucan. No signal was detected when the outer pectin layer remained intact (right panel). Scale bar = 10 μm applies to all images.

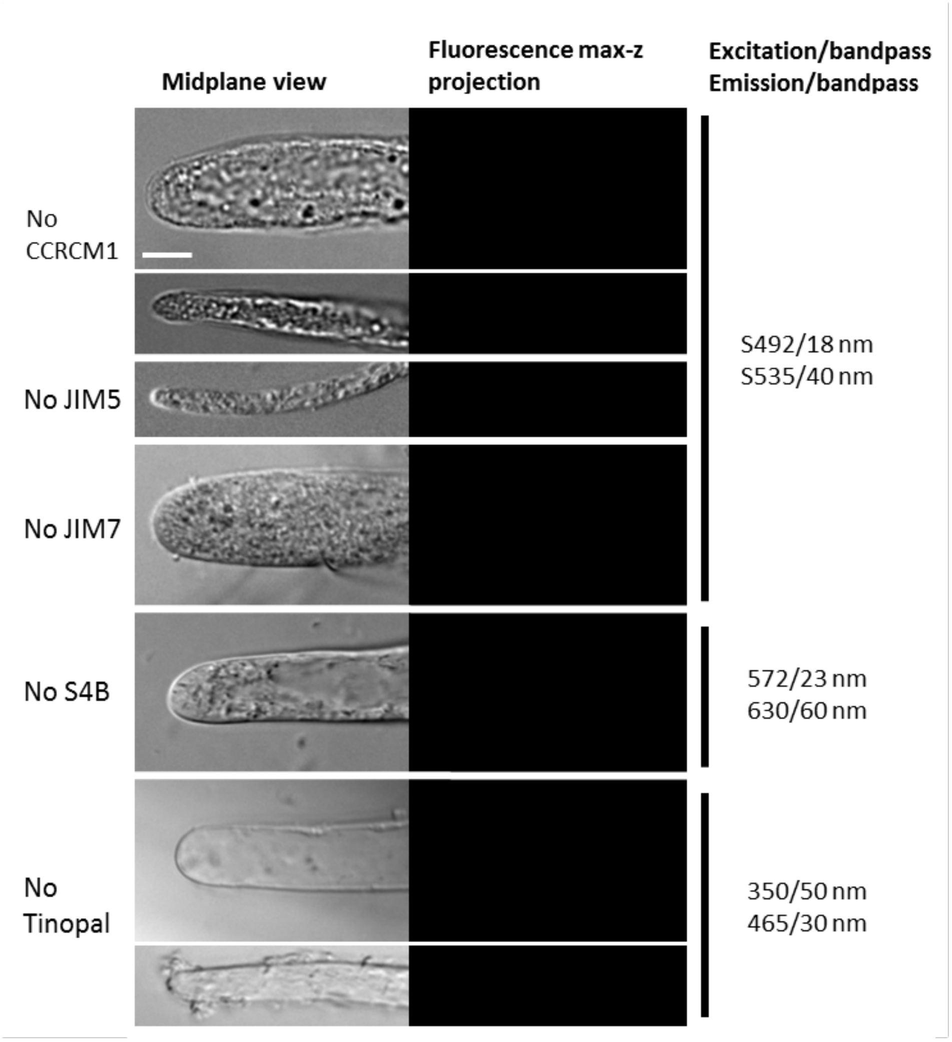

Supplementary Figure S4. No autofluorescence was detected in negative controls where the antibodies or fluorophores were omitted. The left panel shows the fiber tip, and the right panel shows the absence of fluorescence in the maximum Z-projection of the corresponding fluorescence images. Sample preparation was as described in Materials and Methods, except the primary antibodies (CCRC-M1, JIM5, or JIM7), S4B, or Tinopal were omitted. Images were exposed, normalized, and processed equivalently to the positive signals (see **Figures 3, 4, 6** in the main text). Therefore, our data interpretations are based on specific fluorescence without any detectable contribution of autofluorescence. Scale bar = 10 µm for all images.

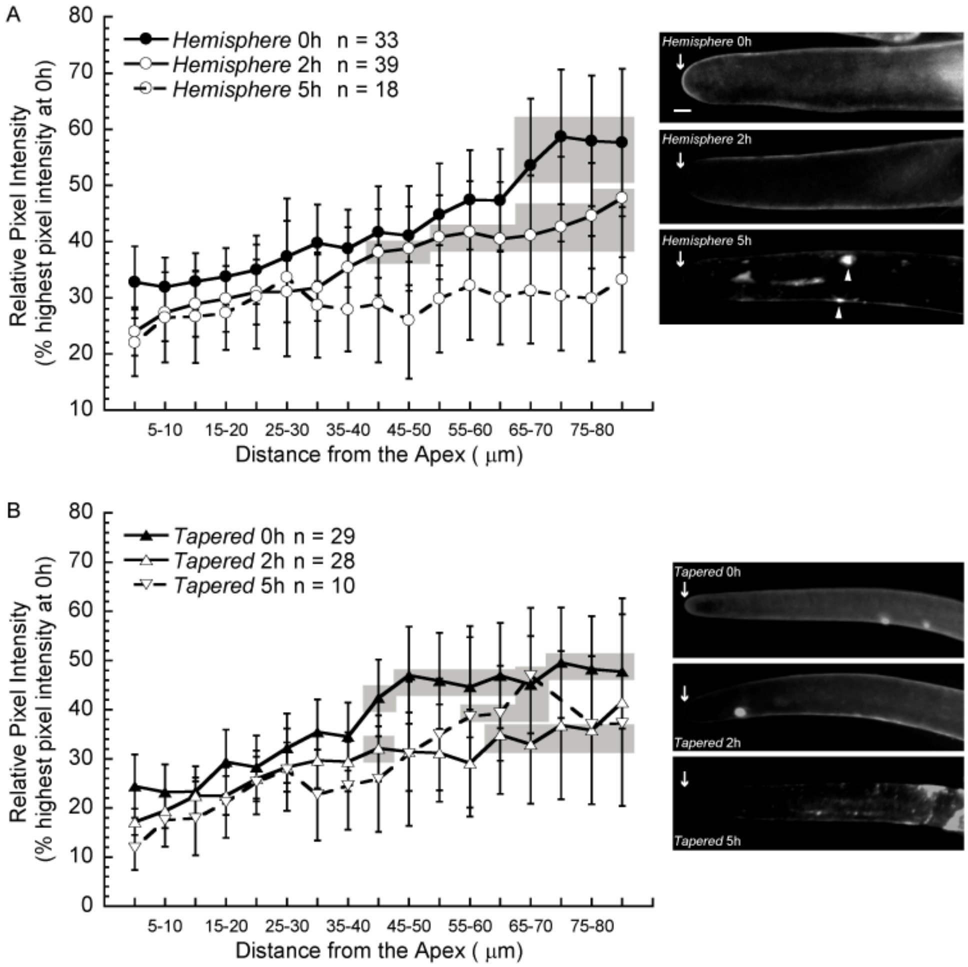

Supplementary Figure S5. Tinopal LPW fluorescence for two *Gh* tip types at 0, 2, and 5 h after pulse-labeling. The data in **Figure 5** were reanalyzed to show the statistically significant differences in relative pixel intensity at distal regions of the tip as compared to the apex at each observation time (0, 2, or 5 h). The significantly different means within each line are marked by grey boxes, with *P*-values indicated below. Images show maximum Z-projections of representative tips, as repeated from **Figure 4**. *Hemisphere* tips (A): At 0 h, relative pixel intensity at 65 – 85 µm was 1.6 – 1.8-fold greater than the apex ($P < 0.007$). After 2 h, the region with greater (1.6 – 2-fold) fluorescence as compared to the apex spanned 40 – 85 µm ($P < 0.03$). After 5 h, the *hemisphere* tips had similar fluorescence in all the regions measured. *Tapered* tips (B): At 0 h, relative pixel intensity at 40 – 85 µm was 1.7 – 2-fold greater than the apex ($P < 0.02$). After 2 h, the 40 – 45 µm and 60 – 85 µm regions showed 1.9 – 2.4-fold greater intensity ($P < 0.04$) than the apex. After 5 h, pixel intensity from 55 – 70 µm ($P < 0.03$) was 3.2 – 3.9-fold greater than the apex. Scale bar = 5 µm applies to all images. Arrowheads indicate spots of bright CFML, which were readily distinguished from the fiber cell wall. Regions like this were excluded from quantitative analyses. All images were acquired under the same conditions and intensity data were normalized to the highest pixel intensity at 0 h for either *hemisphere* (A) or *tapered* tips (B). Values were graphed at the midpoint of the region measured. Error bars represent 95% confidence intervals. Statistical analysis of the means in each graph—distal regions as compared to the apex—was performed with ANOVA and Dunnett's posthoc test.

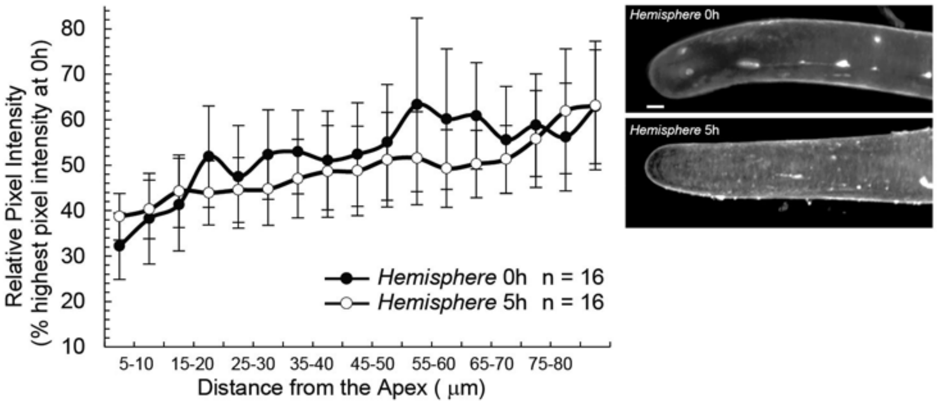

Supplementary Figure S6. Cell wall fluorescence intensity at the tips of dead fibers remains constant for five hours after pulse-labeling with Tinopal LPW. All procedures were performed as described for **Figure 5** of the main text except that, after pulse-staining and rinsing, the living 5 DPA *Gh* fibers were treated with 0.02% (w/v) sodium azide for 10 min to kill the cells. The images show maximum Z-projections of representative *hemisphere* tips imaged immediately (0 h) or incubated *in vitro* (as dead tissues) in the absence of the fluorophore for 5 h before imaging. The graph shows that the relative pixel intensity at the apex or successive distal regions did not change significantly at 5 h as compared to 0 h ($P > 0.07$, as determined by T-tests for each region). Both the 0 h and 5 h graphs for dead cells were similar to the data for living fibers imaged at 0 h (see text **Figure 5**). These dead-control data show that the fluorophore does not spontaneously diffuse away from the cell walls of aerial fibers during 5 h after staining. Therefore, lower fluorescence values in living cells at 2 h or 5 h (**Figure 5**) are reasonably explained by active cell wall synthesis that does not occur in dead cells. Scale bar = 5 µm applies to both images. All images for measurement were acquired under the same conditions and intensity data were normalized to the highest pixel intensity at 0 h within the fiber tip population. Values were graphed at the midpoint of the region measured. Error bars represent 95% confidence intervals.

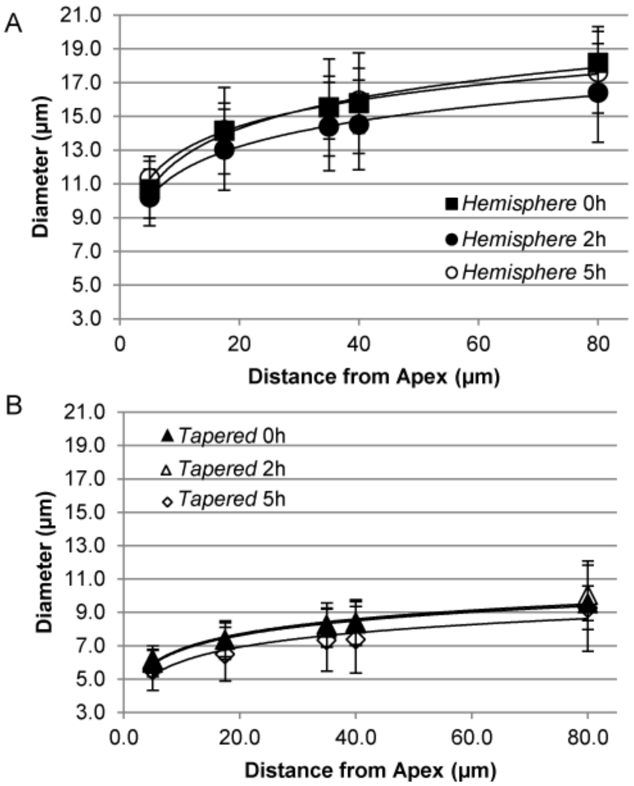

Supplementary Figure S7. Tip diameters did not change during 5 h *in vitro* growth after Tinopal pulse-labeling. Diameters were measured at intervals behind the apex to determine whether there was any diametric expansion that could explain the decreases in fluorescence that occurred in the absence of the fluorophore. (A) The diameter of *hemisphere* tips averaged 12.34 and 17.39 µm diameter at 10 and 80 µm from the apex, respectively. (B) *Tapered* tips averaged 6.54 and 9.61 µm diameters at 10 and 80 µm from the apex, respectively. The same fibers analyzed in text **Figure 4** were measured. (n = 10 – 39 fiber tips). Trend lines represent logarithmic regressions (R^2 = 0.893 - 0.998). Error bars represent standard deviations. The mean apical diameters at 10 µm are close to those in Table 1, and the small differences likely relate to the measurements being made on fixed (Table 1) or living fibers (this figure).

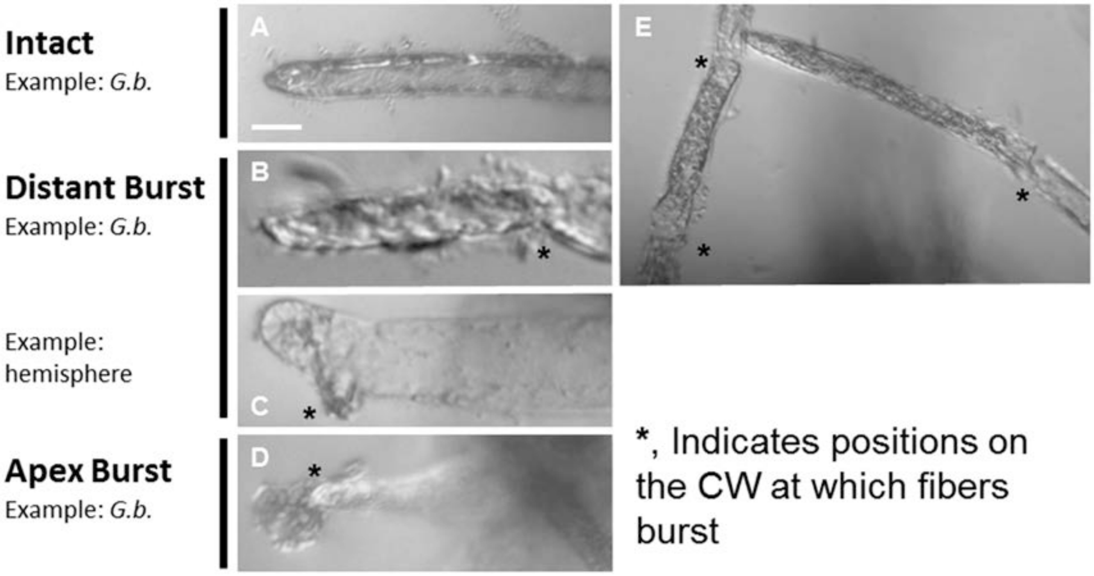

Supplementary Figure S8. Cell wall digestion of living fibers revealed regions of weaker cell wall (CW). Living fibers were incubated (5 min, RT) in BT medium containing 1 U/mL of a cell wall-degrading enzyme mixture, predominantly pectinase (P2401, Sigma Aldrich), prior to fixation in HistoChoice®. Illustrating the quantitative information in the main text, *Gh hemisphere*, *Gh tapered*, and *Gb* fibers were scored as Intact (A; no ruptures), Distant Burst (B & C; ruptures > 5 µm from the apex), or Apex Burst (D; ruptured at the apex). Ruptures were also observed farther from the apex in some fibers after cell wall digestion (E), but not when incubated with heat denatured enzyme or without enzyme. These distant ruptures were not characterized further since they were outside the tip region. Scale bar = 10 µm applies to all images.

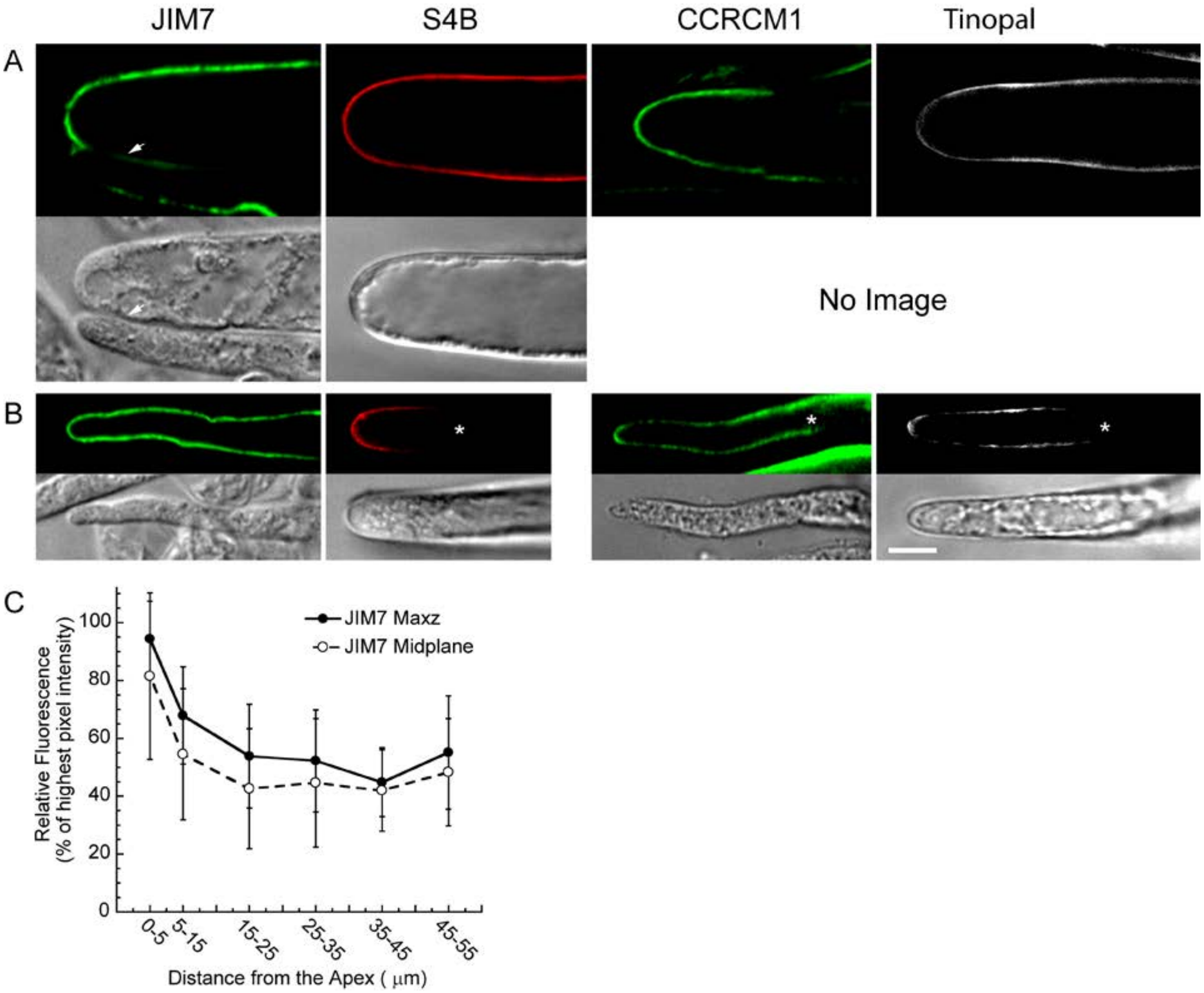

Supplementary Figure S9. Fluorescent probes for cell wall components did not penetrate into the cytoplasm of intact *Gh* fiber tips. (A) Midplane views of *hemisphere* tips. For each of the probes (JIM7, S4B, CCRC-M1, and Tinopal) the fluorescent signal formed a relatively continuous and thin peripheral line. In contrast, thicker cytoplasm with an undulating inner edge is visible in the paired differential interference contrast (DIC) images for the JIM7 and S4B probes. Further emphasizing the unstained cytoplasm, the lower edge of the hemisphere fiber in the JIM7 fluorescent image is dimly stained (arrow) due to the poor penetration of the probe into the cell wall between two attached fibers, although cytoplasm is present in this region. No light micrographs were available for the CCRC-M1 or Tinopal *hemisphere* tips shown. (B) Midplane views of *tapered* tips. As shown by the paired DIC images, these fiber tips are densely filled with cytoplasm, yet the signals from the cell wall probes form a relatively continuous and thin peripheral line. Asterisk (*) indicates portions of the fiber that left the focal plane, leading to no fluorescence (S4B and Tinopal) or visualization of fluorescence on more of the fiber surface (CCRC-M1) remote from the apex. (C) Relative fluorescence in JIM7 labeled *hemisphere* tips was similar when measured from maximum Z-projections (JIM7 Maxz) or from the midplane of the fiber (JIM7 Midplane). Means were derived from n = 9 fibers for each type of measurement. T-test ($\alpha = 0.05$) showed that the means for the two measurement methods at each position were not significantly different. Scale bar = 10 μm.

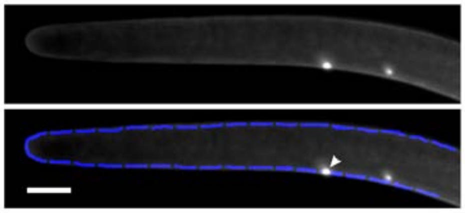

Supplementary Figure S10. Measurements of pixel intensity were made at the apex and at regular intervals in the cell wall along the perimeter of fiber tips. In this example, a *Gh tapered* tip was stained with Tinopal LPW (upper panel) and measurement regions (lower panel, blue) were defined around the perimeter of the apex (ending 5 µm behind the apex) and at 5 µm intervals in the distal region. The measurement regions were defined with the small diameter pencil tool (0.63 µm width). Measurement regions that included brightly fluorescent patches of CFML (arrowhead) were not included in the quantitative analyses. Scale bar = 10 µm.
